# G-CSF, rt-PA and combination therapy after experimental thromboembolic stroke

**DOI:** 10.1186/2040-7378-2-9

**Published:** 2010-04-14

**Authors:** Rainer Kollmar, Nils Henninger, Christian Urbanek, Stefan Schwab

**Affiliations:** 1 Department of Neurology, University of Erlangen, Erlangen, Germany; 2 Department of Neurology, University of Massachusetts Medical School, Worcester, USA; 3 Neurology Hospital, Ludwigshafen, Ludwigshafen, Germany

## Abstract

**Background:**

Granulocyte Colony-Stimulating Factor (G-CSF) has remarkable neuroprotective properties. Due to its proven safety profile, G-CSF is currently used in clinical stroke trials. As neuroprotectants are considered to be more effective in the early phase of cerebral ischemia and during reperfusion, G-CSF should to be tested in combination with thrombolysis. Therefore, combination therapy was investigated in an experimental model of thromboembolic stroke.

**Methods:**

Male Wistar rats (n = 72) were subjected to a model of thromboembolic occlusion (TE) of the middle cerebral artery. Different groups (n = 12 each) treated by recombinant tissue-plasminogen activator (rt-PA) or/and G-CSF: group control (control), group early G-CSF (G-CSF 60 min after TE), group rt-PA (rt-PA 60 min after TE), group com (combination rt-PA/G-CSF), group delayed rt-PA (rt-PA after 180 min), group deco (G-CSF after 60 min, rt-PA after 180 min). Animals were investigated by magnetic resonance imaging (MRI) and silver infarct staining (SIS) 24 hours after TE.

**Results:**

Early G-CSF or rt-PA reduced the infarct size compared to all groups (p < 0.05 to p < 0.01) with the exception of group com, (p = n.s.) as measured by T2, DWI, and SIS. Late administration of rt-PA lead to high mortality and larger infarcts compared to all other groups (p < 0.05 to p < 0.01). Pre-treatment by G-CSF (deco) reduced infarct site compared to delayed rt-PA treatment (p < 0.05). G-CSF did not significantly influence PWI when combined with rt-PA. All animals treated by rt-PA showed improved parameters in PWI indicating reperfusion.

**Conclusions:**

G-CSF was neuroprotective when given early after TE. Early combination with rt-PA showed no additional benefit compared to rt-PA or G-CSF alone, but did not lead to side effects. Pretreatment by G-CSF was able to reduce deleterious effects of late rt-PA treatment.

## 

Granulocyte Colony-Stimulating Factor (G-CSF) is neuroprotective in models of acute experimental cerebral ischemia [1-9]. During the acute phase of ischemic stroke, various neuroprotective effects of G-CSF have been described in different species [1,2,9]. G-CSF influences apoptotic pathways [3,4], suppresses edema formation and interleukin-1 beta expression [1,6] induces the cerebral G-CSF receptor [7], and diminishes glutamate induced neurotoxicity [1,10]. Moreover, reduction of infarct size is associated with an improved functional score [6,8,11]. Remarkably, G-CSF reduced the infarct size even when given 72 hours after induction of cerebral ischemia [8]. In addition, G-CSF stimulates endogenous neurogenesis and vascularisation [6,7,9,11,12]. As a result, clinical studies are currently conducted to test the safety and effectiveness of G-CSF after acute ischemic stroke [9,11,13].

Because of its multiple ways of action and good clinical tolerability for other medical conditions [9], G-CSF might be an ideal drug for the treatment of acute ischemic stroke. So far, thrombolysis by recombinant tissue-plasminogen activator (rt-PA) within the first 4.5 hours after symptom onset is the only proven effective therapy for thromboembolic stroke [14,15]. As recanalisation and neuroprotection are probably the most promising therapeutical approaches in stroke, combination of rt-PA and G-CSF need to be tested experimentally before using it in patient trials. G-CSF might decrease infarct volume when combined with rt-PA, but may interfere with potentially beneficial effects of rt-PA such as improvement of cerebral blood flow (CBF). Additionally, pretreatment by G-CSF might influence infarct volume and overall outcome after delayed and therefore potentially harmful reperfusion. In this context, the interaction of rt-PA associated pathways and simultaneous treatment by hematopoetic growth factors is of high clinical relevance, since combination treatment by erythropoetin (EPO) and rt-PA lead to increased side effects in a recently published randomized study [16]. It is very remarkable that no animal experiments have been published for combination of rt-PA and EPO before starting the clinical study. Therefore, we investigated whether G-CSF was neuroprotective in a model of thromboembolic stroke. Moreover, the combination therapy with rt-PA was evaluated to eludicate whether G-CSF alters infarct growth in combination therapy. Pretreatment with G-CSF before delayed rt-PA administration was investigated to evaluate whether reperfusion associated injury could be influenced.

## Materials and methods

### Animals and experimental groups

The animal experiments were performed after approval of the animal care committee (Regierungspräsidium Karlsruhe, Germany). Before surgery male Wistar rats (n = 72) weighing 280 to 320 g (Charles-River Deutschland, Sulzfeld, Germany) were assigned to one of the following groups.

• ***Group control ****(n = 12): *Thromboembolic cerebral ischemia (TE). No specific treatment.

• ***Group early G-CSF****(n = 12): *TE followed by intravenous G-CSF treatment after 60 min.

• ***Group early rt-PA****(n = 12): *TE followed by intravenous rt-PA treatment 60 min after TE.

• ***Group com****(n = 12): *TE followed by intravenous rt-PA treatment 60 min after TE and intravenous G-CSF treatment 60 min after TE.

• ***Group delayed rt-PA****(n = 12): *TE followed by intravenous rt-PA treatment 180 min after TE.

• ***Group deco****(n = 12): *TE followed by intravenous G-CSF treatment after 60 min and intravenous rt-PA treatment 180 min after TE.

All animals were subjected to MRI monitoring including perfusion weighted imaging (PWI), diffusion weighted imaging (DWI), T2, and T2* at 0.5, 2.5, 4, and 24 hours after TE followed by silver-infarct staining (SIS) as described below.

### Animal preparation

For induction of anesthesia, the animals inhaled a gas mixture of halothane (Forene; Abott, Wiesbaden, Germany), nitrous oxyide (70%) and oxygen (30%) via a precalibrated vaporizer (Fortec; Cyprane Keighley, United Kingdom). The right femoral artery and vein were cannulated using polyethylene catheters (PE-50; Labokron, Sinsheim, Germany). Body temperature was kept constant at 37°C with a temperature-controlled heating pad (Föhr Medical Instruments, Germany) during surgery. The correlation between body temperature, pericranial and intracranial temperature has been shown before [17].

TE was induced as previously described [18]. Briefly, the right common carotid (CCA), internal carotid (ICA), and external carotid artery (ECA) were exposed and further dissection identified the origin of the pterygopalatine artery (PPA). The ECA and the PPA were permanently ligated while the CCA was only temporarily clipped for embolization. A PE 50 catheter was inserted into the ECA proximal to its ligation and 12 red blood clots (each 0.35 mm in diameter and 3 mm in length) were injected at the origin of the right middle cerebral artery (MCA). The whole surgical procedure lasted 30 to 40 min.

### Rt-PA and G-CSF treatment

Rt-PA (Alteplase, Boehringer Ingelheim, Ingelheim am Rhein, Germany) was infused intravenously at a dose of 10 mg/kg body weight (b.w.). Ten percent were given as a bolus at the beginning of thrombolysis followed by continuous infusion over a 30-minute period with a Harvard pump (Harvard Apparatus). The dose was in accordance with experimental studies [18,19]. Rt-PA dissolved with 1 ml of saline 0.9%, while pure saline was administered to the animals in the control group a. Recombinant G-CSF (Neupogen, Amgen, Thousand Oaks) in a dose of 60 μg/kg body weight was dissolved in 1 ml of 0.9% saline and administered over a period of 30 min 60 min after TE. The dose was in accordance with a previous study [1].

### MRI protocol

The animals were examined in a 2.35-T scanner (Biospec 24/40, BRUKER Medizintechnik, Ettlingen, Germany). An actively shielded gradient coil with an inner diameter of 120 cm was used. This coil was driven by the standard 150 V/100A gradient power supply. In this configuration, 180 mT/m could be reached in 180 ms. A home-built birdcage resonator with an inner diameter of 40 mm as RF coil was used. MRI examination started within 30 min after TE and was repeated at 2.5 h, 4 h, and 24 h. The protocol included T2-WI, DWI, T2*, and PWI as described previously [18,20,21]. For PWI, a bolus of 0.5 mmol/kg bw gadodiamide (Omniscan^®^, Amersham, Braunschweig, Germany) was injected at the scan at 30 min, 2.5 hours, and 4 hours.

An investigator blinded for the treatment groups measured the lesion volumes in T2-weighted and DWI-weighted MR images by tracing the area of hyperintense regions. The infarct volume was calculated similar to the method described for SIS staining. The apparent rrCBV was calculated from the PWI data, as previously described [18,20,21]. PWI data was assessed in a region of interest (ROI) with an area of 3 × 3 pixels at the level of the lateral basal ganglia, which represents a typical ischemic region after occlusion of the MCA. Values were then calculated in percent of the healthy contralateral hemisphere.

### Measurement of infarct volume by the silver infarct staining (SIS) method

The SIS method was used to measure infarct size [18,22]. Following the last MRI investigation after 24 hours, the animals were scarified and brain slices were stained according to the SIS method. A modified version of the semi automated method [23] was used to measure the cerebral infarct volume and calculated as follows:

### Functional neurological outcome

All surviving animal were tested for neurological outcome using the neuroscore according to Menzies [24]: 0 = no apparent deficit, 1 = contralateral forelimb flexion; 2 = decreased grip of contralateral forelimb grip while tail pulled; 3 = spontaneous movement in all directions, contralateral circling only if pulled by tail; 4 = spontaneous contralateral circling. The testing was performed by a co-worker who was blinded for the earlier treatment regimen.

### Statistical analysis

Values of the result section and figures are presented as mean ± S.D. However, functional outcome is given as median and range. After acquiring all the data, the randomization code was broken. ANOVA and subsequent post hoc Fisher protected least significant difference tests were used. A value of p < 0.05 was considered statistically significant. Animals that died before the endpoint of 24 hours were not excluded from MRI, but SIS analysis. The nonparametric Kruskall-Wallis test evaluated the neuroscore with subsequent group comparisons by Mann-Whitney *U *test. The Chi square test with Yates correction for small numbers was used to test for differences mortality rate.

## Results

### Physiological variables

There were no significant differences in the physiologic variables (data not shown).

### Survival

Survival rate was 75% for the control group, the early G-CSF group, the early rt-PA group, and the deco group. 83% of the animals survived in com group. In contrast, most animals died in the delayed rt-PA group which was treated by rt-PA 180 min after TE. Only 5 animals survived 24 hours in this subgroup which corresponds to a survival rate of 41%. Therefore, the survival rate was significantly lower compared to all other experimental groups (p < 0.05). Animals that died prematurely were assessed for the cause of death and showed all large infarcts in the brain territory supplied by the MCA. Therefore, large infarct was probably responsible for the death.

### Functional outcome

Functional outcome was as follows: control group showed a median of 2 (range 2-3), the early G-CSF group had a median of 2 (range 1-2) and the early G-CSF group a median of 2 (range 1-2). Animals of the early combination group showed a non significant trend towards a better outcome compared to all other groups, since the median Menzies score was 1 (range 1-2). Animals of the group which received delayed rt-PA treatment had a median score of 3 (range 2-3). Pretreatment with G-CSF lead to a median Menzies score of 2 (1-3).

### Infarct size calculated from SIS after 24 hours

**Figure 1:**
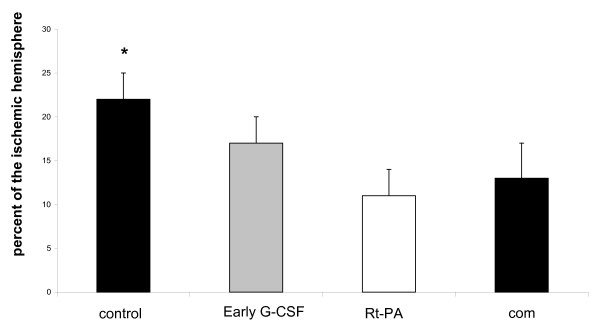
**Infarct volume in SIS**. Cerebral infarct is shown as calculated from SIS after 24 hours. Infarct size is larger in the control group (control) compared to early G-CSF (G-CSF), early rt-PA (rt-PA), and early combination (com) as indicated by the asterisks (p < 0.05).

**Figure 2:**
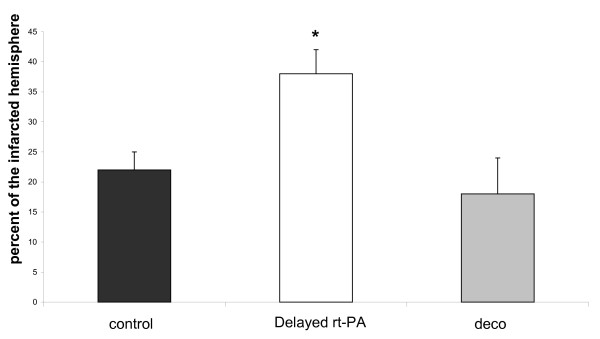
**Infarct volume in SIS after delayed rt-PA treatment**. Cerebral infarct is shown as calculated from SIS after 24 hours. Infarct size is larger in the delayed rt-PA group (delayed rt-PA) compared to the control group (control), and delayed combination (deco) as indicated by the asterisks (p < 0.05).

The extent of cerebral infarction was 38 ± 4% of the right, ischemic hemisphere in the remaining animals of the delayed rt-PA group, 22 ± 3% in the control group, 17 ± 3% in the early G-CSF group, 14 ± 3% in com group, and 11 ± 3% in the early thrombolysis group. Therefore, the infarct size in the delayed rt-PA group was larger compared to all others (p < 0.05). Moreover, the infarct size in the control group exceeded the early G-CSF, early rt-PA group, and com group (p < 0.05). Animals of the deco group had larger infarcts compared to early rt-PA, (p < 0.05). There was no difference between the control group and the deco group (Figure 1 and 2).

### Perfusion weighted imaging

Analysis of the relative regional cerebral blood volume (rrCBV) at the level of the basal ganglia showed a decrease to below 50% of the corresponding nonischemic ROIs 30 min after thromboembolic occlusion (data not shown). This effect was not different between the groups (p = ns). The rrCBV slightly was significantly larger in the rt-PA group and com group compared to all other groups, (p < 0.001). There were no significant differences in the groups not treated by rt-PA (p = ns). 4 hours after stroke onset, rrCBV was larger in the rt-PA group, delayed rt-PA group and the com group compared to the control group and the early G-CSF group, (p < 0.005). There was a significant difference between the rt-PA group (94 ± 11%), delayed rt-PA group (91 ± 7%) compared to the deco group (75 ± 4%; p < 0.05). Data is shown in Table 1.

**Table 1:** Data of serial MRI.

	DWI in mm^3^		
	**2.5 hrs**	**4 hrs**	**24 hrs**

**control**	43 ± 5	86 ± 14	102 ± 11

**early G-CSF**	31 ± 5	65 ± 17	71 ± 8

**rt-PA**	26 ± 7	57 ± 17	68 ± 9

**com**	21 ± 8	49 ± 14	65 ± 11

**delayed rt-PA**	43 ± 4	136 ± 22	180 ± 13

**deco**	33 ± 5	80 ± 14	87 ± 12

	**T2 in mm^3^**		

	**4 hrs**	**24 hrs**	

**control**	79 ± 12	113 ± 7	

**early G-CSF**	65 ± 18	85 ± 8	

**rt-PA**	68 ± 13	69 ± 8	

**com**	54 ± 16	70 ± 15	

**delayed rt-PA**	129 ± 14	193 ± 15	

**deco**	68 ± 13	93 ± 13	

	**PWI in %**		

	**2.5 hrs**	**4 hrs**	

**control**	28 ± 3	58 ± 7	

**early G-CSF**	34 ± 5	53 ± 8	

**rt-PA**	78 ± 4	94 ± 11	

**com**	75 ± 6	91 ± 7	

**delayed rt-PA**	34 ± 3	90 ± 5	

**deco**	38 ± 7	75 ± 4	

These are given as means ± SD for different time points after stroke onset.

### Diffusion weighted imaging

There were no significant differences between the lesion size after 0.5 hours as calculated from DWI (p = ns; data not shown).

**Figure 3:**
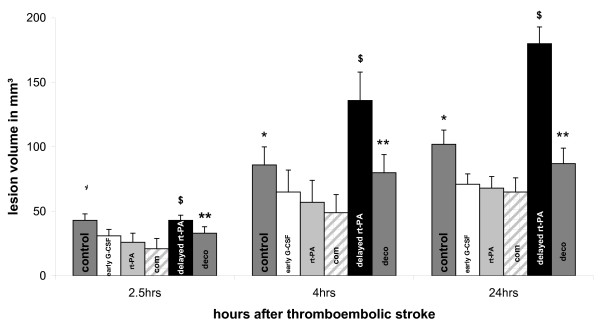
**Lesion volume in MRI**. Lesion volume is shown as calculated from DWI and expressed as means ± SD. The lesion volume in the control group was larger compared to early rt-PA (*rt-PA*) and early combination group (*com*) after 2.5 hours, and larger than the early rt-PA, early G-CSF (*G-CSF*) and early combination group after 4 and 24 hours as shown by the single asterisks. Delayed rt-PA treatment (*delayed rt-PA*) resulted in larger lesion volume than the early rt-PA, early G-CSF and early combination group after 2.5 hours, and additionally larger than the control group after 4 hours. It was larger than all others after 24 hours as shown by the string sign. The late combination group (*deco*) showed larger infarcts than the early combination after 2.5 hours. After 4 hours, lesion volume was larger than all others with exception of delayed rt-PA. After 24 hours, there was a difference compared to the early rt-PA and early combination as indicated by the double asterisks. Levels of significance are given in table 2.

DWI lesions after 2.5 hours accounted to 43 ± 5 mm^3 ^in the control group and were significantly larger than those observed for the rt-PA group by 26 ± 7 mm^3^, and early com group by 21 ± 8 mm^3 ^(p < 0.05), (Data shown in Figure 3). There was no significant difference compared to the early G-CSF group by 31 ± 5 mm^3^, the deco group by 33 ± 5 mm^3^, and the delayed rt-PA group with 42.7 ± 4 mm^3^. Animals treated with early G-CSF tended towards smaller lesions, since the lesion volume was 26 ± 6 mm^3 ^in the early G-CSF group and 21 ± 8 mm^3 ^in the com group. Early thormbolysis lead to a lesion volume of 25.9 ± 9 mm^3 ^(p < 0.05). 4 hours after stroke onset, lesion volume was larger in the delayed rt-PA group (135.8 ± 22 mm^3^) than in the deco group (80.2 ± 14 mm^3^; p < 0.05), the early G-CSF group (65.1 ± 17 mm^3^; p < 0.005), and early rt-PA (57 ± 17 mm^3^; p < 0.005). After 24 hours, the lesion volume of the delayed rt-PA group was 180 ± 13 mm^3 ^and significantly larger than in the control group (102.4 ± 11 mm^3^; p > 0.001), the early G-CSF group (71.1 ± 8 mm^3^; p < 0.001), the deco group (87.3 ± 12 mm^3^; p > 0.001), and the early rt-PA group (68.3 ± 9 mm^3^; p > 0.001). Moreover, lesion volume in the early G-CSF group and the early rt-PA group was smaller than in the control group (p < 0.05).

Values of DWI after 24 hours show significant differences between the treatment groups (Table 1 and 2). The infarct volume was larger in the control group a vs. early G-CSF group (p = 0.019), to early rt-PA group (p = 0.006), and the com group (p = 0.013). No treatment resulted in smaller infarct volume than G-CSF plus delayed rt-PA administration in group deco (p = 0.002). There was a non-significant trend towards smaller infarct volume in group deco compared to the control group (p = 0.25; n.s.). Comparing the early treatment group G-CSF and rt-PA, there was no difference for the infarct volume. Combining rt-Pa and G-CSF (group com) in the early phase lead to smaller infarct than for rt-PA treatment alone (p = 0.001). Early rt-PA treatment (group rt-PA) and combination (group com) lead to smaller infarcts than in group delayed rt-PA (p = 0.001). Pretreatment by G-CSF in combination with delayed rt-PA in group deco reduced the infarct volume compared to the delayed rt-PA group (p = 0.001).

**Table 2:** Comparing diffusion-weighted imaging

after 2.5 hours						
	**control**	**early** **G-CSF**	**rt-PA**	**com**	**delayed rt-PA**	**deco**

**control**		n.s.	< 0.05	< 0.05	n.s.	n.s.

**early G-CSF**	n.s.		n.s.	n.s.	< 0.05	n.s.

**rt-PA**	< 0.05	n.s.		n.s.	< 0.05	n.s.

**com**	n.s.	n.s.	n.s.		< 0.05	n.s.

**delayed rt-PA**	n.s.	< 0.05	< 0.05	< 0.05		n.s.

**deco**	n.s.	n.s.	n.s.	< 0.05	n.s.	

**after 4 hours**						

	**control**	**early** **G-CSF**	**rt-PA**	**com**	**delayed rt-PA**	**deco**

**control**		n.s.	p < 0.05	p < 0.01	< 0.05	n.s.

**early G-CSF**	n.s.		n.s.	n.s.	< 0.005	n.s.

**rt-PA**	p < 0.05	n.s.		n.s.	< 0.005	n.s.

**com**	p < 0.01	n.s.	n.s.		< 0.005	n.s.

**delayed rt-PA**	< 0.05	< 0.005	< 0.005	< 0.005		< 0.05

**deco**	n.s.	n.s.	n.s.	n.s.	< 0.05	

**after 24 hours**						

	**control**	**early** **G-CSF**	**rt-PA**	**com**	**delayed rt-PA**	**deco**

**control**		< 0.05	< 0.05	< 0.05	< 0.001	n.s.

**early G-CSF**	< 0.05		n.s.	n.s.	< 0.001	n.s.

**rt-PA**	< 0.05	n.s.		n.s.	< 0.001	< 0.05

**com**	< 0.05	n.s.	n.s.		< 0.001	< 0.05

**delayed rt-PA**	< 0.001	< 0.001	< 0.001	< 0.001		< 0.001

**deco**	n.s.	n.s.	< 0.05	< 0.05	< 0.001	

Levels of significant difference are given here as calculated from DWI-sequences.

### T2-WI and T2*-WI

There were no significant differences among all groups at the first scan after 30 min (data not shown) and after 2.5 hours (p = ns). After 4 hours, lesion size in the delayed rt-PA group was 129.4 ± 14.4 mm^3 ^and therefore larger than in the early G-CSF group (65.3 ± 18 mm^3^), the com group (68.1 ± 13 mm^3^), and the rt-PA group (58.1 ± 12 mm^3^); (p < 0.005). After 24 hours, infarct size was larger in the control (113 ± 7 mm^3^) compared to the early G-CSF group (85.5 ± 8 mm^3^; p < 0.05), the com group (70.3 ± 15 mm^3^; p < 0.01), and the early rt-PA group (59.1 ± 8 mm^3^); (p < 0.005). However, infarct size in the control group was smaller compared to the delayed rt-AP group (193.3 ± 5 mm^3^; p < 0.001). Moreover, the lesion size in the delayed rt-PA group was larger than in the com group, the early G-CSF group, and early rt-PA group (p < 0.001). T2*-WI showed 4 animals (one in each group excluding early thrombolysis) with an intra-cerebral hemorrhage.

## 4. Discussion

This study shows for the first time that G-CSF reduces infarct volume in a model of thromboembolic stroke. Moreover, it prevents some deleterious effects of delayed rt-PA treatment in terms of infarct growth and mortality during an observation time of 24 hours after stroke onset. These results are an important step towards further clinical investigations on G-CSF and acute stroke, since the EPO trial failed because of deleterious combination therapy of EPO and rt-PA [16].

### Neuroprotective effects of G-CSF

Recent experimental studies showed that G-CSF is beneficial after cerebral ischemia and brain injury [1-8]. While neuroprotective effects are described in the early stage of brain injury, G-CSF also stimulates neuronal progenitor cells providing a link to functional recovery [6,9]. G-CSF reduces infarct volume after transient suture occlusion of the MCA and protects neurons against glutamate-induced excitotoxicity in cell culture [1,10]. Further neuroprotective mechanisms include an increased STAT3 regulation in the penumbra of G-CSF-treated rats. Effects of G-CSF are probably mediated by a special neuronal G-CSF receptor [1,7], since G-CSF passes even the intact the blood-brain-barrier [7] and therefore reaches injured brain regions. Moreover, G-CSF seems to have additional regenerative effects as bone marrow cells are activated. Neuronal plasticity and vascularisation were proven in experimental studies of cerebral ischemia [6,7].

### G-CSF compared to early thrombolysis

So far, G-CSF was not tested in models of thromboembolic stroke. However, this step is essential when transferring neuroprotective agents to stroke patients. If neuroprotectants are successful at all, the chance is the highest in early stages of stroke. In this study, MRI-data as well as SIS showed that G-CSF was as effective as early thrombolysis in terms of reduction of infarct volume. There was no difference in mortality as well. G-CSF did not influence rrCBV compared to the control group. As expected, early rt-PA treatment resulted in almost complete normalisation of rrCBV. It can be suggested that the mentioned multiple neuroprotective effects contribute to reduction of infarct for this subgroup. These results are in accordance to former studies in suture occlusion model [1,6].

### G-CSF prior to delayed thrombolysis

G-CSF reduced infarct volume and mortality when given prior to delayed rt-PA treatment. Moreover, there was no significant difference to early rt-PA and G-CSF treatment alone. In accordance to data of the suture occlusion model, delayed restoration of CBF leads to larger infarct than early restoration. Aronowski et al. observed larger infarct volumes and edema as compared to permanent ischemia, when the MCA occluding suture was removed 120 to 300 min after induction of ischemia [25]. Clinical data shows that delayed thrombolysis beyond the three hours window in already demarked infarct increases the risk of side effects such as hemorrhage or enlargement of infarction [14]. Probably these side effects are caused by reperfusion-associated injury and neurotoxic properties of rt-PA [26,27]. In contrast to the patient, infarct mature much faster in rats and therefore a time window of three hours can be considered as late thrombolysis. In accordance, early rt-PA treatment lead to smaller infarcts compared to the control group, while delayed administration of rt-PA increased infarct volume and mortality in this study. It can be suggested that G-CSF helps to prolong the time window for thrombolysis. This is of importance, since the transport of patients to the clinic is often long and excludes them from thrombolytic treatment. Pre-treatment In the ambulance car or if the treatment with rt-PA is delayed in the hospital might be helpful for these subgroup of stroke patients.

However, there are several limitations of the study. Certainly, observation periods of more than 24 hours are necessary to test whether effects of neuroprotectants are transient or permanent. Moreover, further investigations should address whether G-CSF interacts with rt-PA and CBF. Autoradiographic techniques may answer these questions, but were not in the focus of the present study. Animals were exposed to anesthesia for several hours. While this could interfere with mortality overall, differences between the groups cannot explained with it. Although we did not investigate further pathways of G-CSF and combination with rt-PA, this study is essential when G-CSF will be investigated in patients treated by rt-PA.

In conclusion, the results of the present study are encouraging on the path of new therapies for ischemic stroke. G-CSF represents an interesting and promising candidate for stroke therapy because of its neuroprotective properties, potential induction of stem cells and good clinical tolerance in hematological patients. Further experimental studies have to investigate combination therapy of G-CSF and rt-PA over longer time periods, since combining the so far best medical therapy rt-PA with new drugs represents a logical and potentially successful way for stroke treatment.

## Competing interests

The authors RK and SS are involved into the AXIS-trial investigating safety and feasibility of G-CSF after acute stroke. Moreover, they own a patent on the use of growth-factors such as G-CSF for the treatment of stroke.

## Authors' contributions

RK and SS designed the study, did the statistics and prepared the manuscript. NH and CU performed the experiments. NH investigated neurological examination of the animals. All authors read and approved the final manuscript.
